# Highly Stretchable and Robust Electrochemical Sensor Based on 3D Graphene Oxide–CNT Composite for Detecting Ammonium in Sweat

**DOI:** 10.3390/bios13030409

**Published:** 2023-03-21

**Authors:** Yunzhi Hua, Mingxiang Guan, Linzhong Xia, Yu Chen, Junhao Mai, Cong Zhao, Changrui Liao

**Affiliations:** 1 Shenzhen Institute of Information Technology, Shenzhen 518172, China; 2 Guangdong Laboratory of Artificial Intelligence and Digital Economy (SZ), Shenzhen 518107, China; 3 Key Laboratory of Optoelectronic Devices and Systems of Ministry of Education and Guangdong Province, College of Physics and Optoelectronic Engineering, Shenzhen University, Shenzhen 518060, China; 4 Shenzhen Key Laboratory of Photonic Devices and Sensing Systems for Internet of Things, Guangdong and Hong Kong Joint Research Centre for Optical Fiber Sensors, Shenzhen University, Shenzhen 518060, China

**Keywords:** electrochemical sensor, wearable sensor, ammonium, sweat, Graphene–CNT network, stretchable sensor

## Abstract

Wearable electrochemical sensors have attracted tremendous attention and have been experiencing rapid growth in recent years. Sweat, one of the most suitable biological fluids for non-invasive monitoring, contains various chemical elements relating abundant information about human health conditions. In this work, a new type of non-invasive and highly stretchable potentiometric sweat sensor was developed based on all-solid-state ion-selective electrode (ISE) coupled with poly(dimethylsiloxane; PDMS) and polyurethane (PU). This highly stretchable composite of PDMS-PU allows the sensor to be robust, with the PDMS providing a flexible backbone and the PU enhancing the adhesion between the electrodes and the substrate. In addition, graphene–carbon nanotube (CNT) network 3D nanomaterials were introduced to modify the ion selective membrane (ISM) in order to increase the charge transfer activity of the ISEs, which also could minimize the formation of water layers on the electrode surface, as such nanomaterials are highly hydrophobic. As a result, the sensor demonstrated a wide detection range of NH_4_^+^ from 10^−6^ M to 10^−1^ M with high stability and sensitivity—showing a high sensitivity of 59.6 ± 1.5 mV/log [NH_4_^+^] and an LOD lower than 10^−6^ M. Under a strain of 40%, the sensor still showed a sensitivity of 42.7 ± 3.1 mV/log [NH_4_^+^]. The proposed highly stretchable and robust electrochemical sweat sensor provides a new choice for wearable-device-based personal daily healthcare management beyond hospital-centric healthcare monitoring.

## 1. Introduction

Sweat, one of the most valuable biological fluids for non-invasive monitoring, contains multiple chemical elements that relate abundance information regarding health conditions. The production of sweat can be accomplished through physical activity, body heating, pressure or ionization energy stimulation [1]. Sweat is rich in metabolites (lactic acid, glucose, uric acid and ethanol), electrolytes (Na^+^, K^+^, Cl^−^, Ca^2+^ and NH_4_^+^), trace metal elements (Zn^2+^, Cu^2+^ and Pb^2+^) and a small amount of large molecules (such as proteins and nucleic acids) [2]. Electrolytes such as sodium and ammonium are critical elements in sweat. Levels of ammonia during perspiration are associated with the breakdown of proteins [3]. Ammonium concentrations in sweat also vary when changing from an aerobic to an anaerobic state, which is quite useful for managing daily exercise. Besides this, as liver converts ammonia to urea before its excretion, high ammonia levels in sweat can be used as biomarkers of hepatic disorders such as hepatitis or cirrhosis [4].

Considering that there are more than 100 sweat glands per square centimeter of skin spread all over the body [5], ion-selective electrode (ISE)-based wearable electrochemical sensors can be worn non-invasively on almost any body location, making it ideal for the continuous monitoring of ammonium concentrations in sweat. An ISE-based sensor is an electrochemical sensor that converts the activity of selected ions in a sample solution to a potential output. ISEs contain a thin membrane that allows for the selective binding of a target ion when ions are transported from areas of high concentration to areas of low concentration. These sensors can be placed close to a location where sweat is easily produced and can be analyzed immediately before the analyte biodegrades. However, conventional ISE-based electrochemical sensors are generally huge, cumbersome and robust [6], making them unsuitable for wearable applications, which require the sensors to be small in size, mounted on a flexible substrate and integrated with a wireless system [7].

Wearable ISE-based electrochemical sensors are typically fabricated on flexible and stretchable substrates, which are capable of conforming to the complex human body profile and will not cause physical discomfort [8]. A wearable sodium sensor based on textiles has been demonstrated [9]. Other groups have also developed textile-based pH, NH_4_^+^, K^+^ [10] and Cl^−^ [11] sensors for sweat. However, these textile-based sweat sensors can only attach to some skin regions for monitoring, as the sensor is not entirely flexible. Compared with textiles, stretchable polymer-based substrates are low-cost, highly flexible, easy to fabricate and simple to attach, making them more suitable for fabricating wearable electrochemical sensors [12]. The first reported stretchable polymeric sweat sensor was demonstrated as a tattoo-based sweat sensor [13].

As ammonium concentrations in human sweat can be as low as 10^−4^ M [14], the ISE-based sweat sensor is required to have a low enough limit of detection (LOD), which is related to the charge transfer activity of the ISE. To improve the charge transfer activity, 3D nanomaterials have been introduced into ISE-based sweat sensors due to their much-enhanced surface areas [15], which allow for enhanced charge transfer activity.

In this paper, graphene–carbon nanotube (CNT) network 3D nanomaterials have been introduced to modify the ion-selective membrane (ISM) to increase the charge transfer activity of ISEs. The introduction of the 3D Graphene–CNT network also minimized the formation of a water layer on the electrode surface, as such nanomaterials are highly hydrophobic, which also enhances the accuracy of ammonium detection and long-term stability. In addition, a highly stretchable composite material (e.g., poly-dimethylsiloxane (PDMS)-polyurethane (PU)) was used as the substrate, in which PDMS provided a flexible backbone while PU enhanced the adhesion between the electrodes and the substrate. As a result, the proposed sensor turned out to have a high sensitivity of 59.6 ± 1.5 mV/log [NH_4_^+^] and an LOD lower than 10^−6^ M. Under a strain of 40%, the sensor still turned out to have a sensitivity of 42.7 ± 3.1 mV/log [NH_4_^+^]. We anticipated that this highly flexible and robust sensor could be applied as a personal daily healthcare management solution based on a wearable sensor.

## 2. Materials and Methods

### 2.1. Device Fabrication

**Figure 1:**
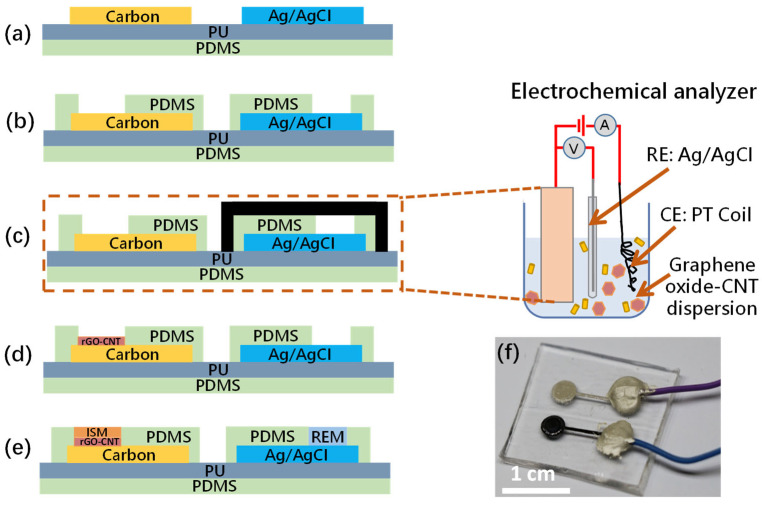
Fabrication process flow of the stretchable electrochemical sweat sensor: (**a**) Screen printing of the Carbon and Ag/AgCl layers on a PU-PDMS substrate; (**b**) Screen printing an additional PDMS layer to act as the electric insulator; (**c**) Electrochemical deposition of the Graphene–CNT layer at −1.22 V using an electrochemical analyzer, with the protection of the screen-printed reference electrode on the substrate by tape; (**d**) the 3D Graphene–CNT layer was formed on the screen-printed carbon electrode; (**e**) Forming the ISM by the drop-casting method; (**f**) The fabricated sensor bonded with wires.

The fabrication process is illustrated in Figure 1. First, to form a stretchable substrate of ~2 mm thickness, a fixed amount of polydimethylsiloxane (PDMS, Sigma-Aldrich, St. Louis, MO, USA) solution was poured into a tape-bounded cavity on a glass plate. The PDMS layer was partially cured in a vacuum chamber at 120 °C for 50 s to avoid bubble formation. This half-cured state facilitated adhesion between the hydrophobic PDMS and the additional 150 µm-thick hydrophilic polyurethane (PU, BASF, Mannheim, Germany) layer. The PU was prepared by dissolving 15 wt% PU in dimethylformamide (DMF, Sigma-Aldrich, St. Louis, MO, USA) solution and pouring into the partially cured PDMS cavity. This was followed by curing at 50 °C for 30 min. The total thickness of the PDMS/PU substrate was ~2.15 mm. As shown in Figure 1a, the screen-printing pattern was drawn using AutoCAD (Autodesk, San Rafael, CA, USA) and the electrodes were printed onto the PU substrate with ~70 μm thickness. A separate stencil pattern was used for each layer (working electrode, reference electrode and insulator). The working electrode was formed by screen printing carbon ink (Zhongyi Inks Company, Zhongshan, China) with 10 wt% PDMS on the PU substrate and was cured at 120 °C for 20 min. The reference electrode was formed by screen printing Ag/AgCl ink (Dupont, Wilmington, NC, USA) with 10 wt% PDMS and was cured at 120 °C for 6 min. Then, as shown in Figure 1b, another PDMS layer was screen printed as an insulator layer and was cured at 120 °C for 30 min to form cavities, acting as the sensing areas for the active and reference electrodes.

Cyclic voltammetry (CV) was used to determine the reduction voltage peak of the Graphene oxide (GO) solution (the setup is shown in Figure S1). Graphene oxide (3 mg/mL, Sigma-Aldrich, St. Louis, MO, USA) containing 0.2 M LiClO_4_ (Alfa Aesar, Haverhill, MA, USA) solution was used as the electrolyte. To prepare the 3D Graphene–CNT network, 0.3 mg/mL of MWCNTs were added to the electrolyte and then sonicated for 30 min to obtain a homogeneous dispersion. In the CV result shown in Figure S1, a large cathodic peak was observed at about −1.22 V (vs. Ag/AgCl), which was attributed to the reduction of oxygen functional groups on the GO nanosheets. However, there was no anodic wave in the reverse scan, indicating a totally irreversible reduction of GO.

After the determination of the reduction voltage peak of the GO solution, the electrodeposition of the Graphene–CNT was carried out using chronoamperometry under a constant potential of −1.22 V for 300 s on a carbon electrode. As shown in Figure 1c and Figure S2, during the electrodeposition, the screen-printed reference electrode was protected by tape. To achieve a stable electrochemical reaction, commercial reference and counter electrodes were applied during the deposition. In order to complete the reduction of the remaining GO after electrodeposition, the 3D Graphene–CNT was placed in a reduction solution containing 1 g vitamin C in 100 mL DI water at 40 °C for 2 h [16]. The modified carbon electrode was washed repeatedly with DI water to remove the unabsorbed GO sheets and was preserved in DI water to prevent the collapse of the 3D network that may be caused by drying in ambient conditions. The freeze dry method was carried out to protect the 3D structure during the drying process [17]. As a result, a Graphene–CNT layer with a 3D structure was formed on the screen-printed carbon electrode, as shown in Figure 1d.

The ammonium-selective membrane, constituting the ISM in Figure 1e, was formed by drop-casting on the active electrode film with a solution composed of 1 mg nonactin (Sigma-Aldrich, St. Louis, MO, USA), 66.8 mg bis(2-ethylhexyl) sebacate (DOS, Sigma-Aldrich, St. Louis, MO, USA) and 32.2 mg PU mixed in 1 mL tetrahydrofuran (THF, Sigma-Aldrich, St. Louis, MO, USA). The use of PU was to enhance the mechanical strength of the ISM [18]. The reference electrode membrane (REM) drop-casting solution was prepared by dissolving 78.1 mg polyvinyl butyral (PVB, Sigma-Aldrich, St. Louis, MO, USA) and 50 mg NaCl in 1 mL methanol [3]. Both the ISM and REM were dried overnight at room temperature. Eventually, the fabricated sensor was bonded with wires, as shown in Figure 1f. The sensor works in a two-electrode structure without a counter electrode, so the output is the open-circuit potential generated by the working and reference electrode.

### 2.2. Cyclic Voltammetry (CV)

A three-electrode system was used in the CV test setup using the AC Impedance technique (CHI 660D, CH Instruments, Austin, TX, USA), with the modified carbon electrode as the working electrode, platinum wire as the counter electrode, and a Ag/AgCl electrode as the reference electrode. The testing temperature was room temperature (~25 °C), and the electrolyte was 0.1 M NH_4_Cl. For comparison, the CV tests were performed using a bare carbon electrode, a 2D graphene-coated electrode and a Graphene–CNT-coated electrode, respectively, to evaluate the performance of the sweat sensor with these electrodes.

### 2.3. Electrochemical Impedance Spectroscopy (EIS)

The AC Impedance technique (CHI 660D, CH Instruments, Austin, TX, USA) was also used to determine the electrochemical impedance spectra for the sensor. To investigate the ion-to-electron transducing process, a sine waveform was superimposed onto the base potential and its frequency was scanned from high to low. The impedimetric detection was conducted at an AC power of 100 mV and a frequency range of 0.1 Hz to 1 M Hz. The electrochemical behavior of the ISEs with different modification were characterized by EIS to further reveal the operating mechanisms of the signal transduction and the performance of the ISEs modified by different methods.

## 3. Results

### 3.1. Characterization of Graphene–CNT Composites

**Figure 2:**
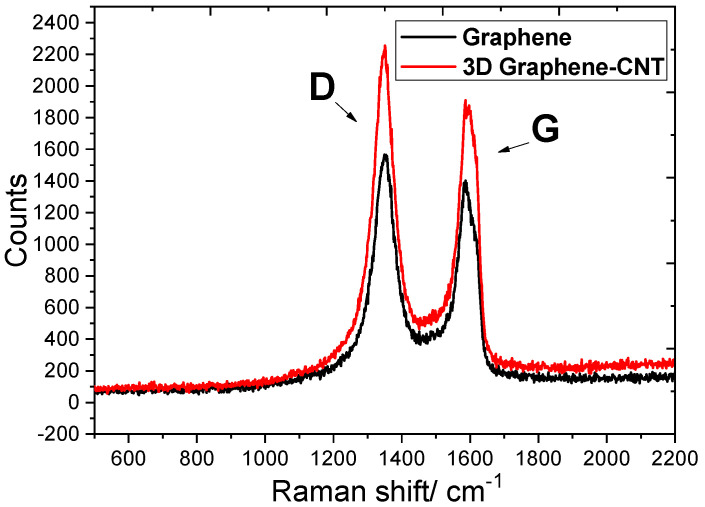
Raman spectra of Graphene network (I_D_/I_G_ = 1.1) and 3D Graphene–CNT network (I_D_/I_G_ = 1.2) with characteristic D and G peaks.

Figure 2 shows the comparison of the Raman spectra between the 3D graphene and the 3D Graphene–CNT network. The Raman spectrum of both spectra include the D-band at 1310 cm^−1^ attributed to an sp2 hybrid carbon atom vibration, associated with graphene edges or some disorder in the graphene structure, and the G-band at 1586 cm^−1^—attributed to the in-plane vibration of the sp2 carbon atoms.

After dropping the active electrode with the Graphene–CNT electrode into the vitamin C reduction solution, the intensity ratio of the I_D_/I_G_ of the spectral peak’s D-band and G-band changed from 1.1 (Graphene network) to 1.2 (3D Graphene–CNT network). The increase in this ratio was due to the reduction of oxygen-containing functional groups in the GO and the increase of surface defects in the graphene, leading to topological disorder. The larger the ratio, the greater the degree of defects, allowing better ion or electron conductance with a better cycle efficiency of the electrodes. This shows that the 3D Graphene–CNT is a better candidate for electrode modification, offering higher ion transfer efficiency for the sweat sensor.

**Figure 3:**
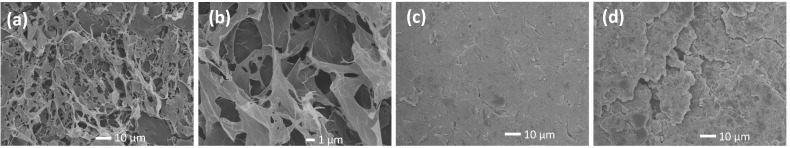
SEM micrographs of the working electrodes with different coatings: (**a**) Electrodeposited 3D GO–CNT layer (**b**) is the zoomed-in micrograph; (**c**) Bare carbon electrode; (**d**) 2D graphene.

Figure 3a shows the field emission scanning electron microscope (SEM) JSM-6390 (JEOL, Peabody, MA, USA) micrographs of the fabricated, highly porous 3D Graphene–CNT network on the active electrode surface, created by applying a cathodic potential of −1.22 V for 300 s to GO aqueous solution (3 mg/mL) containing 0.2 M LiClO_4_. From the zoomed-in SEM micrograph shown in Figure 3b, a porous graphene sheet structure was clearly observed. As a comparison, there was almost no 3D structures on the bare carbon electrode, as shown in Figure 3c. It has also been confirmed that there were much less porous 3D structures on the electrode surface with drop-casted 2D graphene, as shown in Figure 3d. As the crumpled and porous reduced graphene sheets provided a much larger surface area to facilitate the access of electrolytes and target analytes to the active electrode surface, the 3D Graphene–CNT electrodeposited electrode could enhance the surface reaction and ionic transfer during ammonium detection.

**Figure 4:**
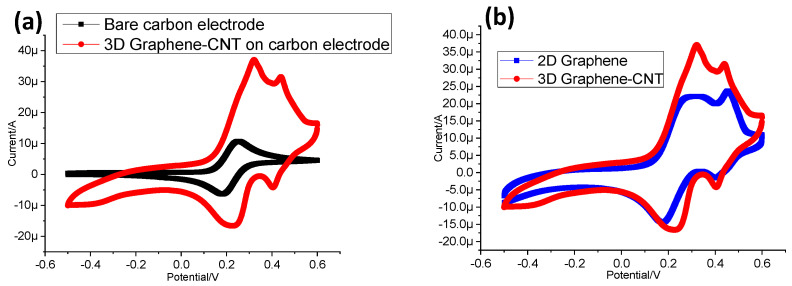
Comparison of the electrochemical surface area of the 3D Graphene–CNT-deposited electrode with that of the (**a**) bare carbon electrode and (**b**) 2D Graphene-deposited electrode, respectively.

To further confirm the increased surface area induced by the 3D Graphene–CNT network on the active electrode surface, the microscopic area of electrodes modified using different methods was characterized by CV in 5.0 mM [Fe(CN)_6_]^3−/4−^ containing 0.1 M NH_4_Cl solution as redox probes. Figure 4 plots the anode and cathode peak currents (I_p_) of the bare carbon electrode and the 2D Graphene and 3D Graphene–CNT deposited electrodes, repectively, at a sweep rate of 200 mV/s. The Graphene–CNT modified electrode responded to a higher current value than that of both the bare carbon electrode (Figure 4a) and the 2D Graphene electrode (Figure 4b). Based on the quantitative comparison of the peak currents, it turned out that the effective surface area of the Graphene–CNT-deposited electrode increased by 40% compared with that of the 2D Graphene and was even 1.7-times higher than that of the bare carbon electrode. The higher peak current is related to the higher effective electroactive surface area of the 3D Graphene–CNT-deposited electrodes, due to the formation of a large number of porous graphene and CNT structures on the electrode surface that were able to significantly increase the number of accessible active sites; it therefore facilitated the charge transfer of the sensor during detection.

### 3.2. Interface Properties of Surface-modified Electrodes

**Figure 5:**
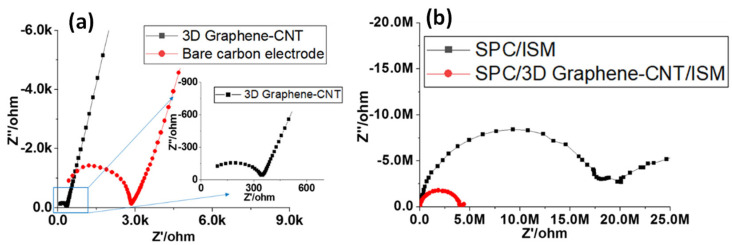
(**a**) EIS results for the bare carbon electrode and 3D Graphene–CNT modified electrode in 0.1 M NH_4_Cl solution, respectively; (**b**) EIS results for the screen-printed carbon (SPC) electrode modified by Graphene–CNT and an NH_4_^+^ ISM coating and that with only an NH_4_^+^ ISM, respectively, in 0.1 M NH_4_Cl solution. (*ΔE*_ac_: 100 mV; frequency range: 0.1 Hz–1 M Hz).

The properties of electrodes with different types of modified surfaces were examined by EIS in 0.1 M NH_4_Cl solution. The results of the EIS are illustrated as a Nyquist plot in Figure 5, in which the semicircular portion observed at the higher frequencies corresponds to the electron transfer-limiting process, while the linear portion at the lower frequencies relates to the diffusion-controlled process. The diameter of the semicircular portion can be regarded as an indicator of the electron transfer resistance (*R_ct_*).

In Figure 5a, the EIS results for the 3D Graphene–CNT modified electrode showed a reduced diameter of the semicircle compared to that of the bare carbon electrode, suggesting that the surface of the 3D Graphene–CNT modified electrode exhibited a lower electron transfer resistance and an increased electron transfer rate. In addition, the steeper slope at lower frequencies of the 3D Graphene–CNT modified electrode indicated a higher double layer capacitance. Figure 5b plots the EIS results of the different electrodes coated with an ion-selective membrane (ISM) made of NH_4_^+^, in which the ions move into the ISM phase and electrons travel through the Graphene–CNT layer to reach the electrode. The reduced diameter of the semicircle on the EIS of the 3D Graphene–CNT modified electrode with ISM also indicates a lower *R_ct_* compared with that of the bare carbon electrode with the ISM. Thus, a more effective ion-to-electron transduction has been demonstrated using high-surface-area carbon materials, e.g., 3D Graphene–CNT. The EIS results were also fitted and verified by the equivalent circuit model (See Supplementary Materials Figure S3 and Table S1), in which the value of the *R_ct_*—with regards to the 3D Graphene–CNT modified electrode with the ISM (~5.8 × 10^6^ Ω)—was much reduced compared to that of the bare carbon electrode with the ISM (~1.47 × 10^7^ Ω).

### 3.3. Ammonium Ion Detection

An ISE exhibits selectivity because the chosen ionophore has a much larger free energy of complexation with the specific target ion than with other ions [19], as shown in Figure S4. With the specific molecular structure of the ionophore, the target ion NH_4_^+^ can “size-fit” the complex and combines with the chosen ionophore perfectly. On the contrary, the wall-like ionophores prevent the formation of both wrapping complexes of crown ether with small ions and complexes with large ions.

**Figure 6:**
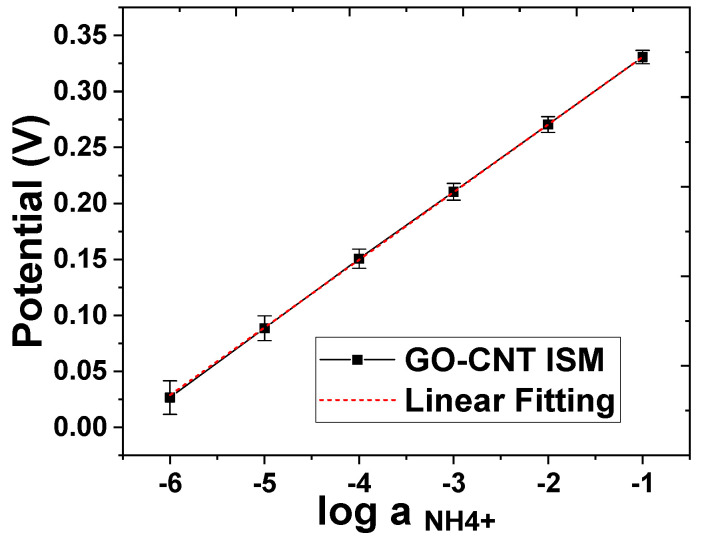
The potentiometric response to a series of testing solutions with NH_4_^+^ concentrations from 10^−6^ M to 10^−1^ M using a sensor with working electrodes modified by Graphene–CNT with an NH_4_^+^ ISM coating.

The potentiometric technique was employed to characterize the performance of the sweat sensor in ammonium ion detection. In this detection, both the working electrode and reference electrode were screen printed. The potential difference between the working and reference electrode was recorded using the Open Circuit Potential–Time (OCPT) technique using a CHI600D analyzer. In the tests using the NH_4_Cl solution with different concentrations from 10^−6^ M to 10^−1^ M, the sensor exhibited an almost ideal Nernstian response, with a sensitivity of 59.6 ± 1.5 mV/log [NH_4_^+^] (2.82 % RSD, *N* = 3) and an LOD lower than 10^−6^ M—as depicted in Figure 6. The testing range covered the typical concentration levels of ammonium ions seen in human sweat [20].

**Figure 7:**
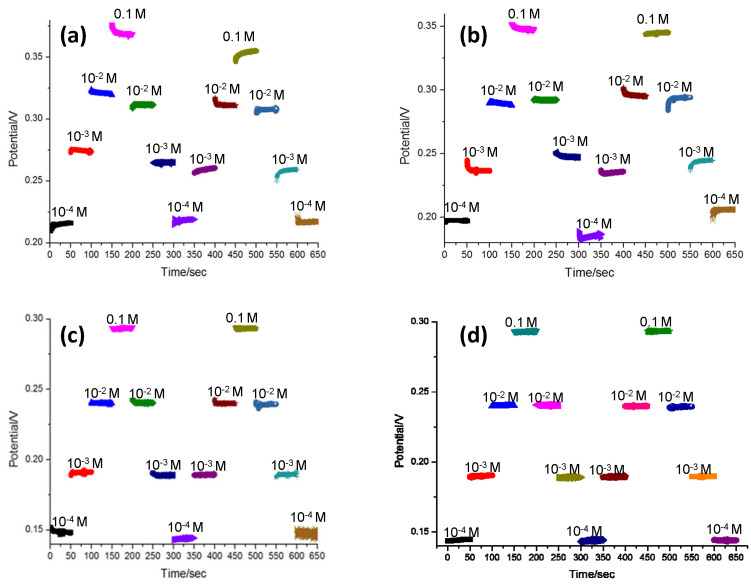
The reversibility of the potentiometric NH_4_^+^ detection using sensors (**a**,**b**) without and (**c**,**d**) with 3D Graphene–CNT modified electrodes, respectively.

To test the reversibility of the potentiometric detection of NH_4_^+^ using the Graphene–CNT modified electrode, the sensors with the two types of electrodes (with and without the Graphene–CNT coating) were dipped into the same set of NH_4_Cl solutions for 50 s, at concentrations varying from 10^−4^ M to 0.1 M. The tests were conducted for two cycles of concentration variation. The potential signal was also recorded by the OCPT technique using the CHI600D analyzer. Figure 7 shows the potential reversibility testing results of the sensors based on the bare carbon electrode and the Graphene–CNT modified electrode. As shown in Figure 7a,b, the potential output of the sensor with the unmodified carbon electrode was not stable for the measurement of each NH_4_^+^ concentration. In addition, the potential signal was also not consistent between the two cycles of measurement. As shown in Figure 7c,d, the sensor with the modified Graphene–CNT electrode exhibited more stable potential measurement results for each NH_4_^+^ concentration, with reduced potential variation between cycles. This comparative experiment demonstrated that the sensor with the modified Graphene–CNT electrode achieved higher reversibility, which is one of the critical performance metrics of wearable sensors. Such enhanced reversibility was probably attributed to the hydrophobic properties of the 3D Graphene–CNT layer (contact angle = 101.53°, Figure S5), which prevents the formation of an aqueous layer between the ISM and working electrode interfaces [21].

**Figure 8:**
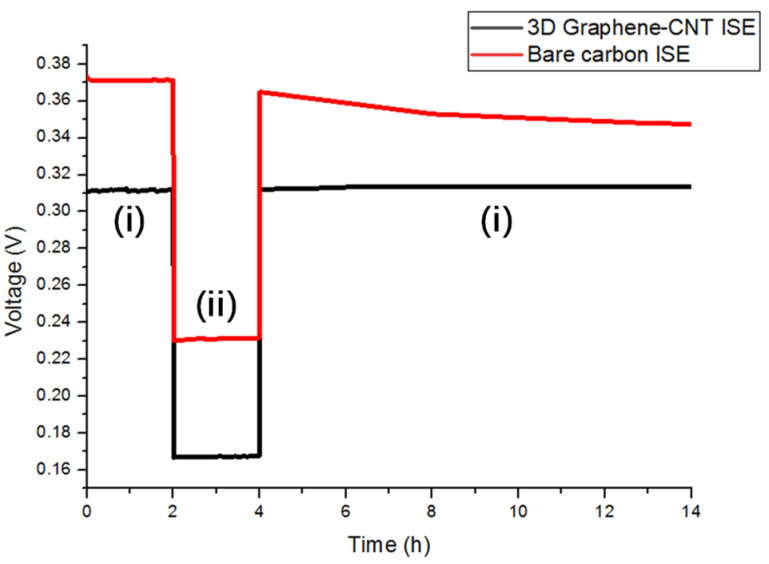
Water layer test results for the 3D Graphene–CNT ISE and bare carbon ISE, determined in (**i**) 0.1 M NH_4_Cl and(**ii**) 0.1 M NaCl.

In order to confirm the presence of an aqueous layer between the ISM and carbon electrode layer, the potentiometric water layer test was carried out according to the following steps [22]: First, the electrodes were immersed in a 0.1 M NH_4_Cl solution of the primary ion for 2 h. After this, the testing solution was changed to 0.1 M NaCl for another 2 h. At the end, the solution was changed back to the initial solution of 0.1 M NH_4_Cl. The potential of the electrodes was continuously monitored during the whole process. As shown in Figure 8, there was a clear drift in the output potential of the sensor with only the bare carbon electrode, which was attributed to the formation of a water layer [23]. Under the same conditions, the sensor with the 3D Graphene–CNT electrode exhibited a stable potential output. This result thus illustrates that stability of the 3D Graphene–CNT electrode, which likely minimizes the formation of an aqueous layer between the ISM and the carbon electrode layer. This result also suggests that the hydrophobic properties of the proposed ISM could effectively minimize the leaking of ISM components, which enhances the stability of the sensor’s potential output.

### 3.4. Stretchability Test

**Figure 9:**
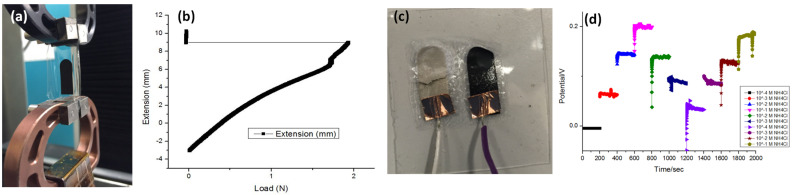
(**a**) The stretched sweat sensor with the PDMS substrate during the tensile test; (**b**) The extension-load curve of the sweat sensor; (**c**) The poor adhesion between the electrodes and the PDMS substrate after the tensile test; (**d**) Potentiometric output of the sensor with the PDMS substrate after the tensile test.

A stretchability test of the sweat sensors based on a PDMS and PDMS/PU substrate was conducted via the tensile test using an UTM-RT05 machine, as shown in Figure 9a. Figure 9b plots the extension-load curve of the sweat sensor with the 3D Graphene–CNT modified electrode on a PDMS substrate’ cracks started to appear on the substrate at 20% strain, and the sample totally broke off at 54% strain. Figure 9c shows the poor adhesion of the sensor after the tensile test to the PDMS substrate. The potentiometric output of the sweat sensor after the tensile test is shown in Figure 9d, with significant fluctuations for different NH_4_Cl concentrations over the two measurement cycles.

**Figure 10:**
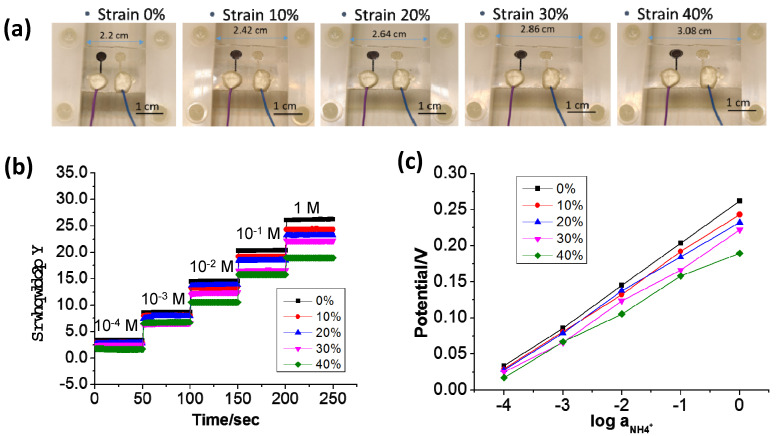
(**a**) Photographs of the 3D Graphene–CNT sweat sensor at different strains (0–40%); (**b**) Potentiometric output of the sweat sensor under a series of NH_4_Cl solutions with different concentrations (10^−4^ M to 1 M); (**c**) Averaged potentiometric output as a function of the concentration of NH_4_^+^.

Figure 10a shows the photographs of the 3D Graphene–CNT modified sweat sensor under different levels of strain (0% to 40%) on the PU/PDMS substrate, on which the sensor still adhered well—although with certain deformations. Figure 10b plots the potentiometric output as a function of NH_4_^+^ concentrations from 10^−4^ M to 1 M under different levels of strain. Compared with the potentiometric output shown in Figure 8d of the sensor with only the PDMS substrate, that of the sensor on the PU/PDMS substrate still looked stable—even at a strain of as high as 40%. Figure 10b plots the potentiometric output of the sweat sensor under a series of NH_4_Cl solutions with different concentrations (10^−4^ M to 1 M), in which the output signal looks very stable. Figure 10c plots the curves of the averaged potentiometric output as a function of the concentration of NH_4_^+^. The curves are generally linear, even under the highest strain level of 40% (Min *R*^2^ = 0.9108). Under a strain of 40%, the sensor still turned out to have a sensitivity of 42.7 ± 3.1 mV/log [NH_4_^+^]. The stretchability test provides evidence that the Graphene–CNT ion-selective membrane is structurally stronger than the bare carbon ion-selective membrane, which could still work stably under a 40% strain—indicating that leakage issues are not significant here.

## 4. Conclusions

A non-invasive and stretchable potentiometric sweat sensor was proposed to measure the ammonium concentration in human sweat. This wearable sensor was based on highly stretchable composite material substrate of PDMS and PU. With modifications of the 3D nanomaterials of the Graphene–CNT network on the ISEs, the charge transfer activity of the ISEs was enhanced and the water-layer issue was minimized, respectively. This highly stretchable and reliable sensor turned out to have a high sensitivity of 59.6 ± 1.5 mV/log and an LOD lower than 10^−6^ M. Under a strain of 40%, the sensor still turned out to have a sensitivity of 42.7 ± 3.1 mV/log [NH_4_^+^]. Additional selectivity studies are critical for the proposed sensor for application in the detection of ammonium in real sweat samples. Thus, our future work is to conduct selectivity tests using samples made of a mixture of NH_4_^+^ and other ions in sweat, such as Na^+^, K^+^, etc. At the final stage, we shall conduct a test by recruiting volunteers to wear our sensor and detect the NH_4_^+^ in their sweat. If further integrated with a wireless communication system, this next-generation sensor could be applied as a key component of a wearable monitoring device used for daily healthcare management.

## Data Availability

The experimental data is contained within the article.

## Supplementary Materials

The following supporting information can be downloaded at: https://www.mdpi.com/article/10.3390/bios13030409/s1, Figure S1: Cyclic voltammetry (CV) for determining the reduction voltage peak of Graphene oxide (GO) solution (3 mg/mL) containing 0.2 M LiClO_4_; Figure S2: Detailed modification process of the Graphene–CNT layer: (a) Key parameters; (b) Setup; Figure S3: (a) Schematic diagram of the layer structure and interfaces in the sensing process, (b) equivalent circuit model (ECM); Table S1: Key parameters in ECM for EIS measurements; Figure S4: Mechanism of ion selectivity based on the specific molecular structure of the ionophore; Figure S5: Contact angle test results of (a) screen-printed bare carbon and the (b) Graphene–CNT modified surface.
