# Supplementary material for: Highly Stretchable and Robust Electrochemical Sensor Based on 3D Graphene Oxide–CNT Composite for Detecting Ammonium in Sweat
Source: Biosensors (Basel). 2023 Mar 21;13(3):409. doi: 10.3390/bios13030409 (PMC10046566; doi:10.3390/bios13030409)
Supplement: Supplementary file 1 [file biosensors-13-00409-s001.zip › biosensors-2266875-supplementary.pdf]

Supplementary Materials

Highly Stretchable and Robust Electrochemical Sensor Based on 3D Graphene Oxide–CNT Composite for Detecting Ammonium in Sweat

Yunzhi Hua [1], Mingxiang Guan [1], Linzhong Xia [1], Yu Chen [1], Junhao Mai [2], Cong Zhao [2,*] and Changrui Liao [3,4]

1 Shenzhen Institute of Information Technology, Shenzhen 518172, China
2 Guangdong Laboratory of Artificial Intelligence and Digital Economy (SZ), Shenzhen 518107, China
3 Key Laboratory of Optoelectronic Devices and Systems of Ministry of Education and Guangdong Province, College of Physics and Optoelectronic Engineering, Shenzhen University, Shenzhen 518060, China
4 Shenzhen Key Laboratory of Photonic Devices and Sensing Systems for Internet of Things, Guangdong and Hong Kong Joint Research Centre for Optical Fiber Sensors, Shenzhen University, Shenzhen 518060, China
* Correspondence: zhaocong@szu.edu.cn

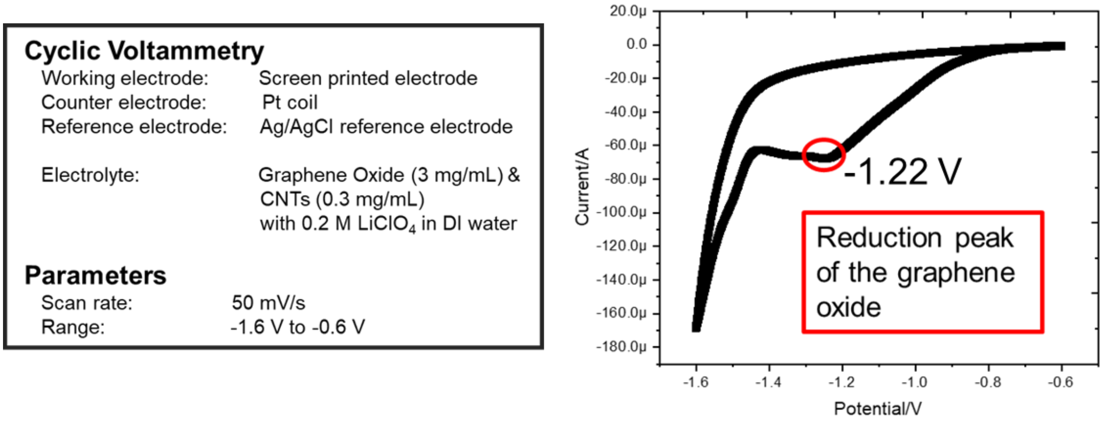

Figure S1. Cyclic voltammetry (CV) for determining the reduction voltage peak of Graphene oxide (GO) solution (3 mg/mL) containing 0.2 M $LiClO_4$.

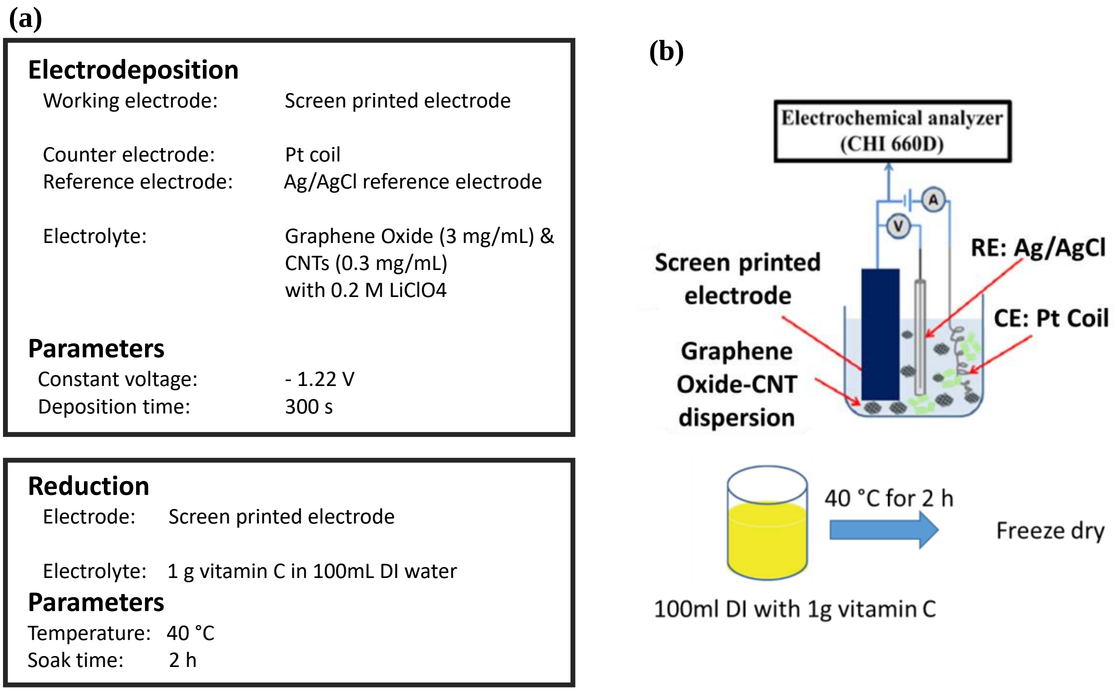

Figure S2. Detailed modification process of the Graphene-CNT layer: (a) Key parameters; (b) Setup.

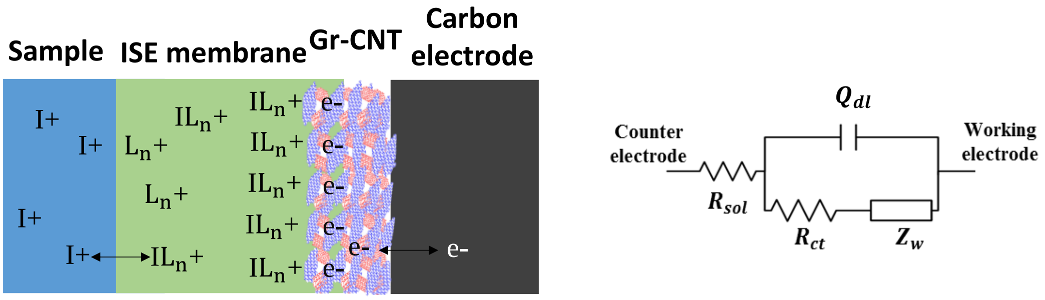

(a) (b)

Figure S3. (a) Schematic diagram of the layer structure and interfaces in the sensing process, (b) equivalent circuit model (ECM).

Table S1 Key parameters in ECM for EIS measurement.

	R_{sol}(Ω)	R_{ct}(Ω)	Z_W (Ω·s$^{-1/2}$)	Q_{dl}(S·s^n)	n
**SPC/ NH_4^+ ISM**	5860	1.47×10^7	3.5×10^7	1.59×10^{-10}	0.802
**SPC/Graphene-CNT/NH_4^+ ISM**	5240	5.8×10^6	2.2×10^7	3×10^{-10}	0.867

R_{sol}: **Solution resistance**, which is unaffected by the target ion

R_{ct}: **Charge transfer resistance**, which is influenced by the energy potential with the complex ion transfer, and it affects the electrostatic and steric energy barrier with the ion passing

Z_W: **Warburg impedance**, which stands for the impedance at low frequencies and it governs the delay arising from the diffusion of redox species to the electrode

Q_{dl}: **Nonideal electrochemical double layer capacitance**, which describes the quantity of charge formed in the electrical double layer

n: index, which refelects the perfection of **Q_{dl}**

Electrochemical Mechanism for the Selective Detection of NH_4^+ Cations

An ion-selective electrode (ISE) based sensor is an electrochemical sensor that converts the activity of the selected ions in a sample solution to a potential output. NH_4^+ ISE contains a thin membrane which achieve the selective binding of a target ion NH_4^+ when ions are transported from the high concentration end to the low concentration end. The ISE usually consists of the following components: (1) Ionophore, (2) common matrix polymer (e.g. PVC), (3) plasticizer (e.g. NOPE, DOS, DOA, etc.) and (4) ionic site (e.g. ETH 500, KTFPB, etc.)

The samples analyzed by ISE contain a number of ions. To obtain a film to selectively absorb and combine with the target ions NH_4^+, the NH_4^+-selective membrane is doped with NH_4^+ ionophores which combine with the target ions NH_4^+ to form reversible complexes. The NH_4^+ ionophores act as the sensing part of the sensor and determine the selectivity of NH_4^+ ISE. In theory, ionophores need to be lipophilic to the plasticized polymer matrix and must strongly but reversibly bind with the target ions.

An ISE exhibits selectivity because the chosen ionophore has a much larger free energy of complexation with the specific target ion than the other ions [1], as shown in Figure S4. With the specific molecular structure of the ionophore, the target ion NH_4^+ can "size-fit" the complex and combines with the chosen ionophore perfectly. On the contrary, the wall-like ionophores prevent the formation of both a wrapping complex of the crown ether with a small ion and complex with a large ion.

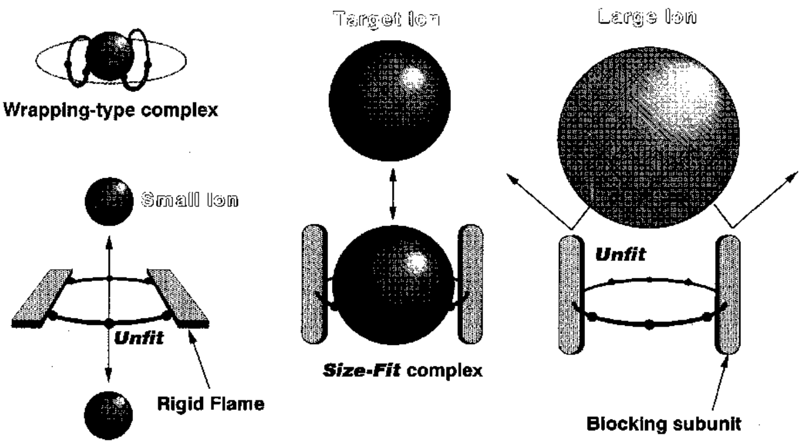

Figure S4. Mechanism of ion selectivity based on the specific molecular structure of ionophore [1]

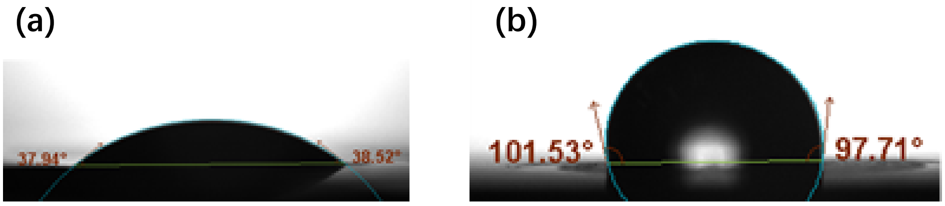

Figure S5. Contact angle test results of (a) screen-printed bare carbon and (b) Graphene-CNT modified surface.

References:

1. Suzuki, K.; Siswanta, D.; Otsuka, T.; Amano, T.; Ikeda, T.; Hisamoto, H.; Yoshihara, R.; Ohba, S. Design and Synthesis of a More Highly Selective Ammonium Ionophore Than Nonactin and Its Application as an Ion-Sensing Component for an Ion-Selective Electrode. *Anal. Chem.* **2000**, *72*, 2200–2205. https://doi.org/10.1021/ac9911241.
